# Complete chloroplast genome of *Tetragonia tetragonioides*: Molecular phylogenetic relationships and evolution in Caryophyllales

**DOI:** 10.1371/journal.pone.0199626

**Published:** 2018-06-22

**Authors:** Kyoung Su Choi, Myounghai Kwak, Byoungyoon Lee, SeonJoo Park

**Affiliations:** 1 Division of Forest Biodiversity, Korea National Arboretum of the Korea Forest Service, Pochen, Republic of Korea; 2 Plant Resources Division, National Institute of Biological Resources of Korea, Incheon, Republic of Korea; 3 Department of Life Sciences, Yeungnam University, Gyeongsan, Gyeongbuk, Republic of Korea; National Cheng Kung University, TAIWAN

## Abstract

The chloroplast genome of *Tetragonia tetragonioides* (Aizoaceae; Caryophyllales) was sequenced to provide information for studies on phylogeny and evolution within Caryophyllales. The chloroplast genome of *Tetragonia tetragonioides* is 149,506 bp in length and includes a pair of inverted repeats (IRs) of 24,769 bp that separate a large single copy (LSC) region of 82,780 bp and a small single copy (SSC) region of 17,188 bp. Comparative analysis of the chloroplast genome showed that Caryphyllales species have lost many genes. In particular, the *rpl2* intron and *infA* gene were not found in *T*. *tetragonioides*, and core Caryophyllales lack the *rpl2* intron. Phylogenetic analyses were conducted using 55 genes in 16 complete chloroplast genomes. Caryophyllales was found to divide into two clades; core Caryophyllales and noncore Caryophyllales. The genus *Tetragonia* is closely related to *Mesembryanthemum*. Comparisons of the synonymous (Ks), nonsynonymous (Ka), and Ka/Ks substitution rates revealed that nonsynonymous substitution rates were lower than synonymous substitution rates and that Ka/Ks rates were less than 1. The findings of the present study suggest that most genes are a purified selection.

## Introduction

Caryophyllales contains 37 families, 749 genera, and 11,600 species [1]. This order is divided into two main clades: core Caryophyllales and noncore Caryophyllales. Previous studies have used molecular phylogenetic analyses based on restriction site data from nuclear and plastid markers [2–4]. DNA sequence data show that the nuclear maker is the ITS (internal transcribed spacer) and the plastid markers are *atpB*, *matK*, *ndhF*, *psbB*, *rbcL*, *rpoC2*, *rps4*, *rpl16* intron, *rpoC1*, IR (inverted repeat) region, and IR junction. Molecular data for Caryophyllales showed this order to be a monophyletic group. However, several molecular studies have shown the phylogenetic position of the Aizoaceae (Tetragonioideae, Aizooideae and Sesuvioideae) within core Caryophyllales [3, 4], and some molecular studies have indicated that Aizoaceae was not included in the core Caryophyllales and was a paraphyletic group [2, 5].

*Tetragonia tetragonioides* (New Zealand spinach) belongs to Tetragonioideae, which is a subfamily of Aizoaceae. *T*. *tetragonioides* is 30–140 cm tall, and its leaves are 3–15 cm long, triangular in shape and thick. It is native to New Zealand, Australia, Chile, Japan, and Korea.

Chloroplast genomes are circular and are typically 120–170 kilobase pairs long. They typically contain a long single copy (LSC) region, a small single copy (SSC) region, and two inverted repeat (IR) regions that typically include ~79 coding genes, 30 tRNA genes, and 4 rRNA genes [6, 7]. Several recent phylogenetic studies have used many chloroplast genes or chloroplast noncoding regions from completely sequenced chloroplast genomes [8–11].

Here, we report for the first time the chloroplast genome of *Tetragonia tetragonioides*. The goals of this study were to (1) present the complete chloroplast genome sequence of *Tetragonia tetragonioides*, (2) compare this sequence with those of other Caryophyllales species, and (3) confirm the phylogenetic and evolutionary relationships within Caryophyllales.

## Materials & methods

The National Institute of Biological Resources (Korea) approved this study. *T*. *tetragonioides* leaves were obtained from Dokdo Island in Korea (37°14′20.97˝, 131° 52′ 6.64˝).

### DNA extraction and sequencing

Total DNA was extracted using a DNeasy Plant Mini Kit (Qiagen Inc., Valencia, CA, USA) and quantified using a HiGenTM Gel & PCR Purification Kit (Biofact Inc., Daejeon, Korea). Genomic DNA was sequenced using Illumina Hiseq 2500 (Illumina Inc., San Diego, CA, USA). A total of 666,785 pair-end sequences read were generated using the DISCOVAR Denovo program, and a total of 863,871 pair-end sequence reads were generated using the Platanus program. A total of 4,142 final contigs were pair-end sequenced at Theragen Co. (Suwon, Korea), and the K-mer length was 17. The resulting contigs were aligned to the *Mesembryanthemum crystallinum* (KM016695) cp genome, which was used for reference purposes. Working primers and additional Sanger sequencing were then used to confirm the four junctions (S1 Table).

### Chloroplast genome annotation and mapping

The complete chloroplast genome sequence was annotated using a DOGMA [Dual Organellar Genome Annotator] [12]. All tRNA genes were verified using corresponding structures predicted by tRNAscan-SE [13]. A circle cp genome map was drawn using OGDRAW [14].

### Repeat structure

REPuter [15] was used to identify the presence of repeat sequences (forward, reverse, palindromic and complementary repeats) in the chloroplast genome of *T*. *tetragonioides*. The following conditions were used to identify repeats in REPtuer: Hamming distance 3, minimum sequence identity of 90% and a repeat size of more than 30 bp. The simple sequence repeats (SSRs) in *T*. *tetragonioides* were detected using Phobos v. 3.3.12 (http://www.ruhr-uni-bochum.de/ecoevo/cm/cm_phobos.htm). Repeats were ≥10 sequence lengths, with three repeat units for mono-, di-, tetra-, and penta-.

### Phylogenetic analysis and substitution rates

Fifty-five gene sequences of 16 species (S2 Table) were aligned using MAFFT [16]. Phylogenetic analysis was conducted by maximum likelihood (ML) using the GTR+R+I model in RAxML v. 7.2.6 [17] and 1,000 bootstrap replicates. To examine the potential link between synonymous substitutions (Ka) and nonsynonymous substitution (Ks), we estimated Ka and Ks rates using alignments of coding genes in Geneious v.6 [18] and analyzed them in DnaSp [19].

## Results

### Chloroplast genome of *Tetragonia tetragonioides*

**Fig 1:**
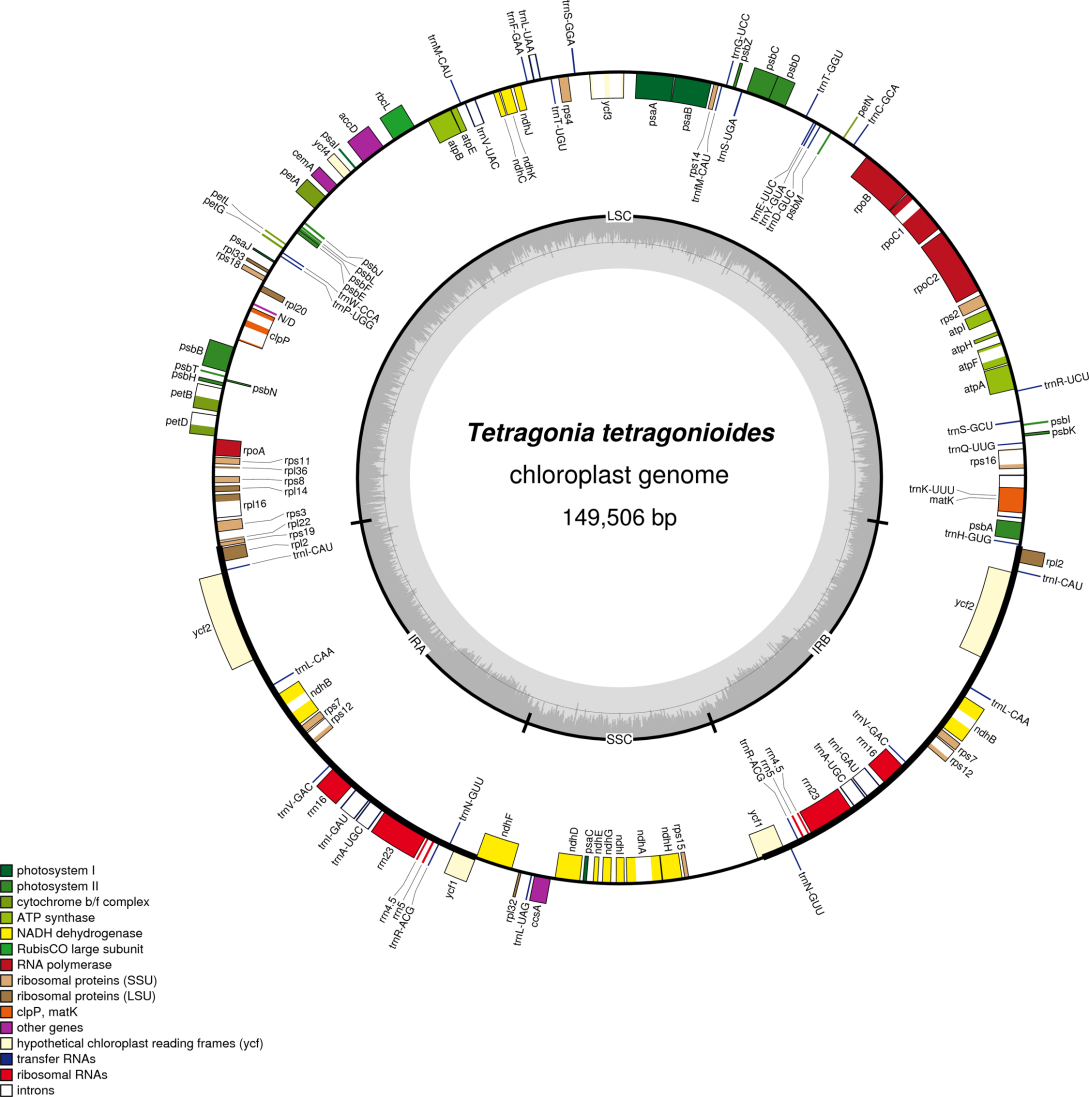
Chloroplast genome of *Tetragonia tetragonioides*. Genes inside the circle are transcribed clockwise, and genes outside are transcribed counterclockwise. The dark gray inner circle corresponds to the GC content, and the light-gray circle corresponds to the AT content.

The cp genome length of *T*. *tetragonioides* was 149,506 bp and contained 82,780 bp in the LSC (large single copy) region, 17,188 bp in the SSC (small single copy) region and 24,769 bp in the IR (inverted repeat) region (Fig 1). The overall AT content of the *T*. *tetragonioides* chloroplast genome was 62.7%, and the AT contents of the LSC, SSC, and IR regions were 64.8%, 69.2%, and 56.9%, respectively.

We identified 110 unique genes in *T*. *tetragonioides*: 77 protein-coding genes, 29 tRNA genes and four rRNA genes. Of the 110 genes, 16 genes contained introns among unique genes of *T*. *tetragonioides*, of which three genes (*clpP*, *ycf3* and *rps12*) included two introns. Seventeen genes in most angiosperm chloroplast genomes have one intron [9, 10, 20]. However, the *rpl2* gene in *T*. *tetragonioides* had no introns.

We analyzed the comprehensive chloroplast genomes of the 17 Caryophyllales species (S2 Table). The cpDNA size of *T*. *tetragonioides* was 149,506 bp, and that of *Carnegiea* (113,064 bp) was shorter than those of other Caryophyllales because *Carnegiea* has lost one IR region and *Drosera* has long IR regions (23,513 bp). The chloroplast genome length of *Tetragonia* (149,506 bp) was shorter than those of other Caryophyllales.

### SSRs and tandem repeats in *T*. *tetragonioides*

**Fig 2:**
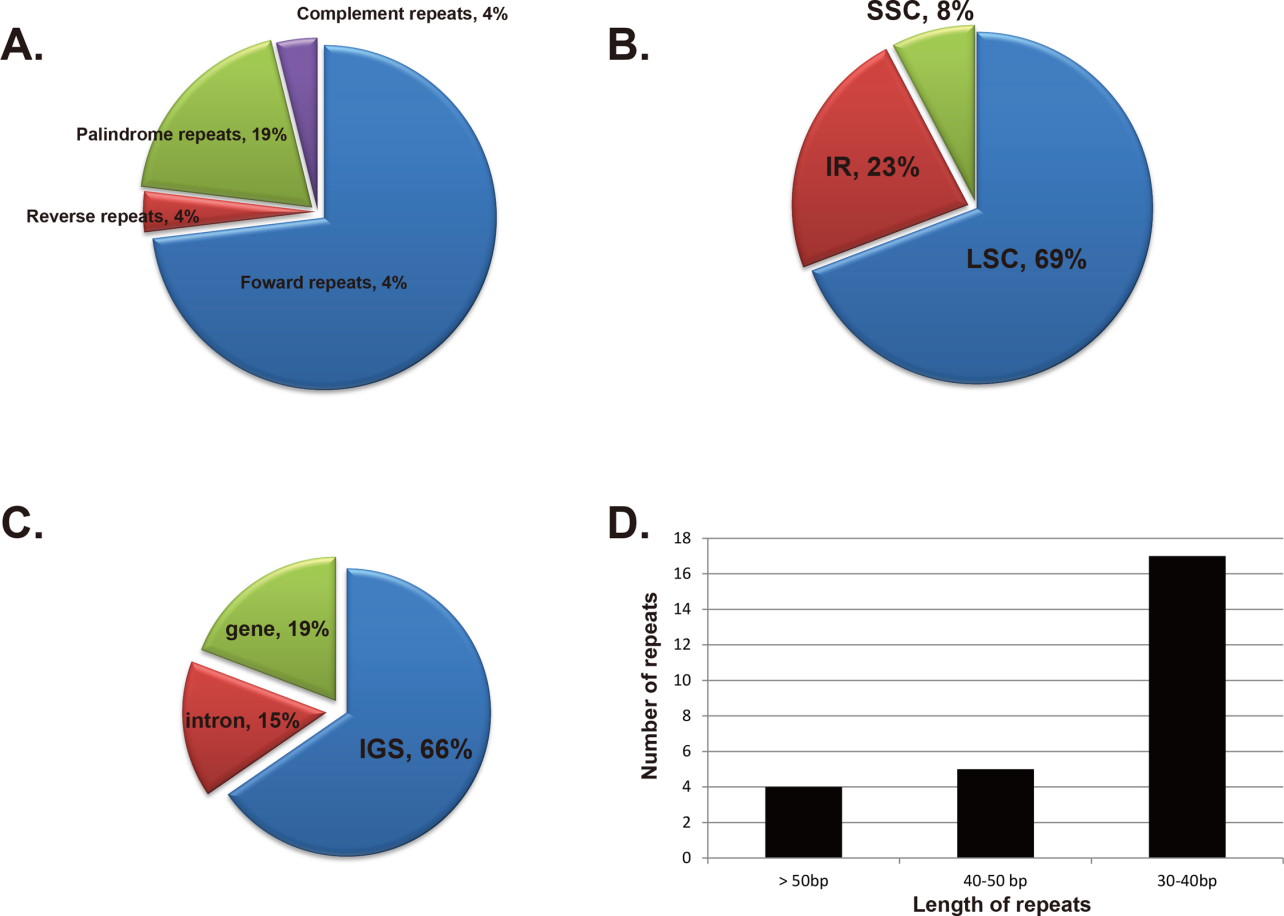
The distribution, types and length of repeats in *T*. *tetragonioides*. A: Number of types of repeats. B: Presence of repeats in the LSC, SSC and IR regions. C: Presence of repeats in protein coding regions, intergenic spacers and intron regions. D: Numbers and length of repeats.

We found forward and palindrome repeats of at least 30 bp long per repeat unit with a sequence identity of ≥ 90%. The results showed that the following were present: 19 forward repeats, 1 reverse repeat, 5 palindromic repeats and 1 complementary repeat (Fig 2). Eighteen repeats were in the LSC region, 6 repeats were in the IR, and two repeats were in the SSC region. Most of the repeats (17) were in intergenic spacers, 4 were in intron region, and 5 were in genes. The longest repeat had a length of 67 bp. Seventeen were 30–40 bp long, 5 were 40–50 bp long, and 4 were 50–67 bp long.

**Fig 3:**
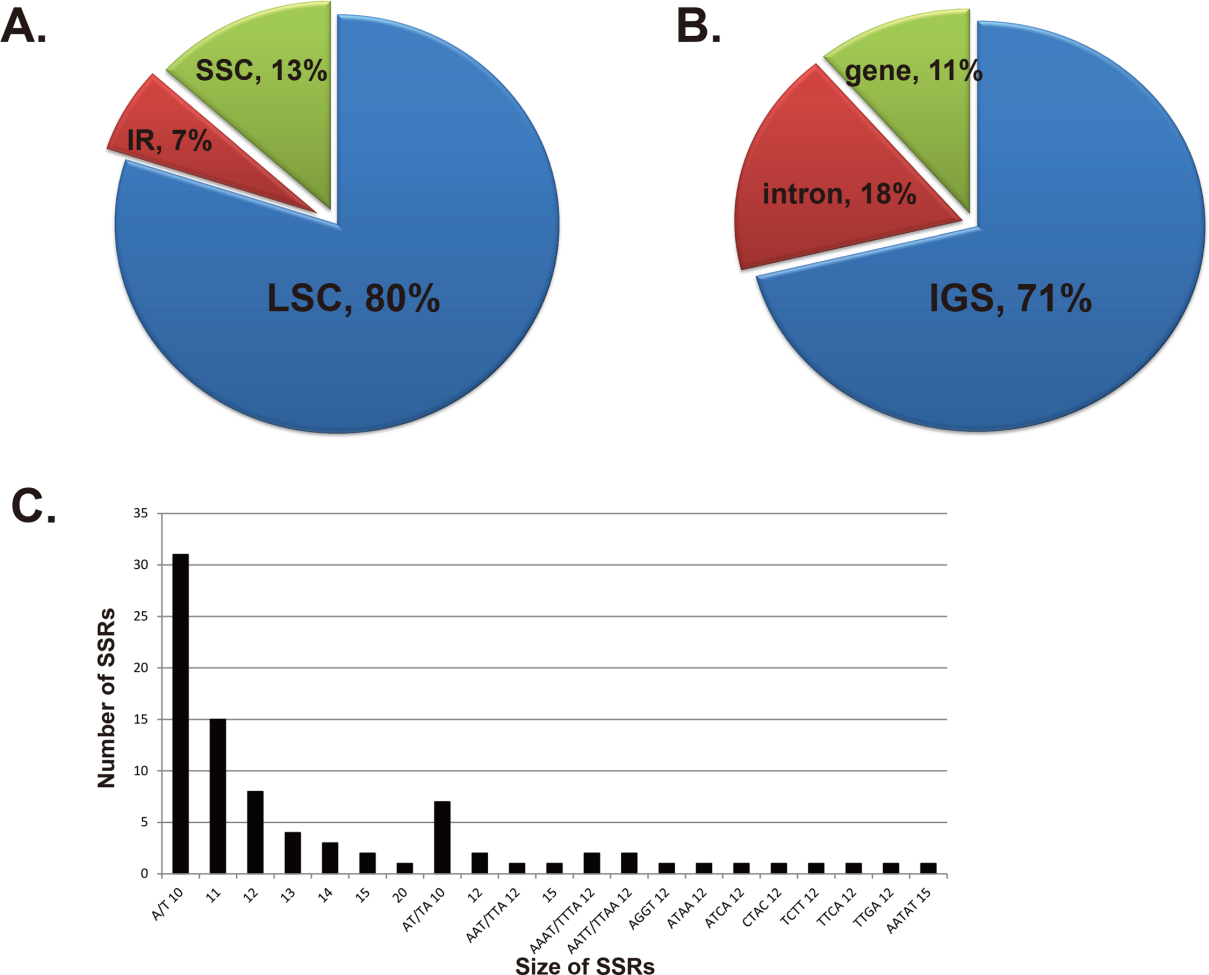
The distribution, types and length of SSRs in *T*. *tetragonioides*. A: Presence of repeats in the LSC, SSC and IR regions. B: Presence of repeats in protein coding regions, intergenic spacers and intron regions. C: Numbers and length of SSRs.

SSRs are highly polymorphic and therefore useful for population genetics. We detected SSRs longer than 10 bp in *T*. *tetragonioides* (Fig 3). The total number of SSRs was 90, and the majority of SSRs were A/T mononucleotides. Most of the SSRs are in the LSC (80%) and are located in intergenic regions (71%).

### Phylogenetic position of *Tetragonia* and Caryophyllales species

**Fig 4:**
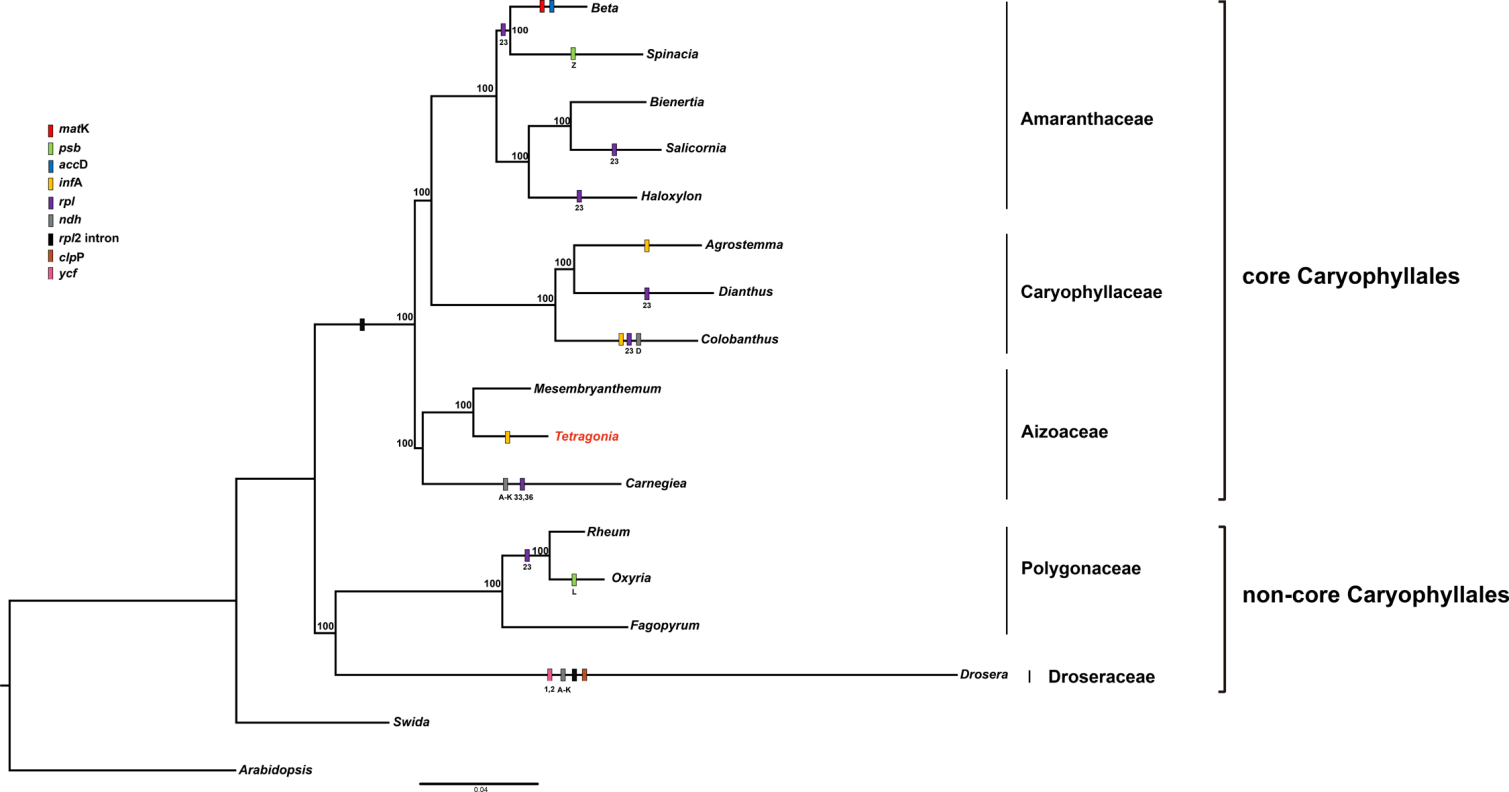
Molecular phylogenetic analyses of 55 protein-coding genes of Caryophyllales. The stability of each tree node was tested by bootstrap analysis with 1,000 replicates. Arabidopsis was used as the outgroup.

Maximum likelihood phylogeny based on 55 chloroplast genes strongly supported the presence of a monophyletic group in Caryophyllales (Fig 4). The two large clades (core and noncore Caryophyllales) were each supported by 100% bootstrap values. Core Caryophyllales (100% bootstrap values) were separated into two groups: the first included *Beta*, *Spinacia*, *Bienertia*, *Salicornia*, *Haloxylon*, *Agrostmma*, *Dianthus* and *Colobanthus*, and the second included *Carnegiea*, *Mesembryanthemum* and *Tetragonia*. *Tetragonia* is sister to the *Mesembryanthemum*. The noncore Caryophyllales group was found to be composed of *Rheum*, *Oxyria*, *Fagopyrum* and *Drosera*.

### *infA* gene and *rpl2* intron loss in *Tetragonia tetragonioides*

Comparisons of Caryophyllales genes revealed many genes that have been lost. Several genes were absent, including *matK*, *psbZ*, *rbcL*, *ycf3*, *accD*, *psbL*, *rps19* and *ndhD* (Fig 4).

**Fig 5:**
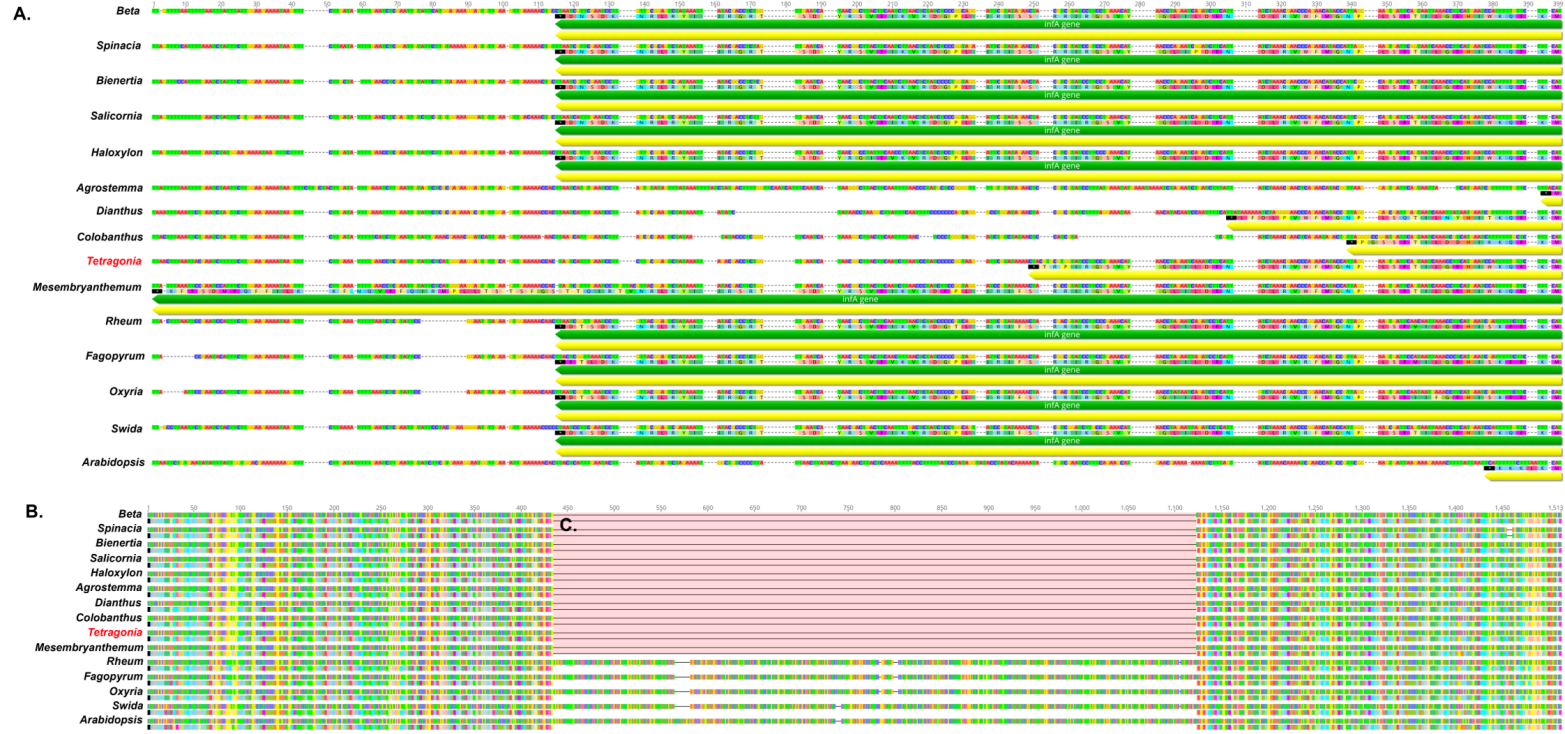
Comparison of *infA* gene and intron of *rpl2* sequences among Caryophyllales. A: *infA* gene region, B: *rpl2* intron region, C: lack of *rpl2* intron. The red word indicates *T*. *tetragonioides*.

The loss of genes in *T*. *tetragonioides* was then analyzed in detail. The *infA* gene was analyzed for 15 species (including 14 Caryophyllales species and *Arabidopsis thaliana*) and found to be a pseudogene or missing in five species, including *T*. *tetragonioides* (Fig 5A). Previous studies indicated that the *infA* gene has been lost in many angiosperms [21]

The chloroplast gene *rpl2*, encoding the large subunit ribosomal protein L2, has one intron (Fig 5B). This study showed that the *rpl2* intron of *T*. *tetragonioides* was absent. Comparisons of Caryophyllales genes revealed many genes that have been lost. In particular, the *rpl2* intron was absent in *Tetragonia* and most Caryophyllales, except three species, that is, *Rheum*, *Oxyria* and *Fagopyrum*, which have been shown to be noncore Caryophyllales in previous studies [3, 4].

### Comparison of substitution rates in Caryophyllales species

**Fig 6:**
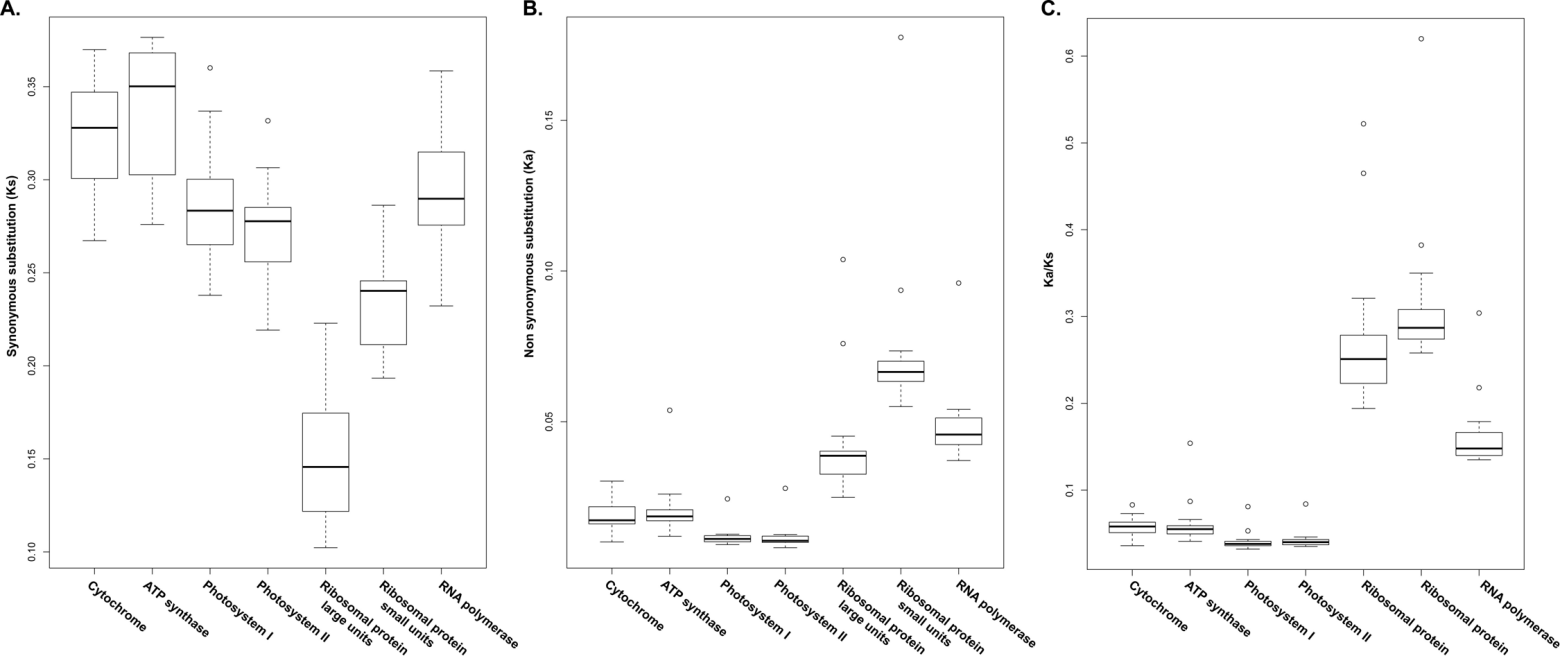
Comparison of nonsynonymous (Ka), synonymous (Ks), and Ka/Ks substitution rates of functional gene groups.

The rates of Ka, Ks and Ka/Ks were compared between Caryophyllales and the *Swida* (Cornaceae, Cornales) to reveal patterns of functional gene evolution (Fig 6 and S3 Table). The Ka values (nonsynonymous substitution) of the cytochrome group, ATP synthase group, photosystem I group, photosystem II group, ribosomal large units group, ribosomal small unit group and RNA polymerase group in Caryophyllales species were approximately 0.018, 0.020, 0.012, 0.012, 0.043, 0.074 and 0.048, respectively. The Ks values (synonymous substitution) of the cytochrome group, ATP synthase group, photosystem I group, photosystem II group, ribosomal large units group, ribosomal small unit group and RNA polymerase group in Caryophyllales were approximately 0.323, 0.339, 0.287, 0.273, 0.152, 0.232 and 0.339, respectively. Average Ka/Ks values ranged from 0.042 to 0.316. The ribosomal protein small unit group had the highest Ka/Ks values, and the cytochrome group had the lowest.

## Discussion

The chloroplast genomes of angiosperms have highly conserved structures and gene orders [10, 19]. However, some angiosperms have lost many genes, and *ndh* genes are pseudogenized in *Erodium* [22] and Orobanchaceae [23, 24], and *infA* and *rpl22* genes are pseudogenized in Rosids [9, 25]. In the present study, comparative analyses of Caryophyllales species chloroplast genomes showed that many genes have been pseudogenized or lost and that some Caryophyllales species (*Drosera* and *Carnegiea*) have lost or exhibited expansion of IR regions.

Downie and Palmer [2] studied the chloroplast genome within Caryophyllales and observed some different gene orders. Caryophyllales demonstrated the loss of the intron of *rpl2* and *rpl16*. Loss of the *rpl2* intron is useful for determining relationships within Caryophyllales [26]. However, our results showed that Polygonaceae, including *T*. *tetragonioides*, has not lost the *rpl2* intron, though our results show that the *rpl2* intron in Caryophyllales has been independently lost multiple times (Fig 4). Moreover, our results regarding the comparative chloroplast genome within Caryophyllales reveal that many have been lost in the chloroplast genome. Previous studies have revealed that many chloroplast genes, such as *infA*, *rpl22*, *rps19*, *rpl2* intron and *rpl23*, are transferred to the nucleus or lost [21, 26, 27]. Our results also revealed several chloroplast gene losses; for example, *Drosera* and *Carnegiea* have lost the *ndh* genes. The chloroplast structures of these two species are quite different, *Drosera* has a long IR region, whereas *Carnegiea* has lost one IR region loss [28].

Previous studies discovered two large subclades within Caryophyllales (core and noncore Caryophyllales) [3, 4]. The present study on 55 combined gene data sets also shows that Caryophyllales is monophyletic and divides into these two large subclades.

According to our analysis, the taxa formerly included in the core Caryophyllales (*Beta*, *Spinacia*, *Bienertia*, *Salicornia*, *Haloxylon*, *Agrostemma*, *Dianthus*, *Colobanthus*, *Mesembryanthemum*, *Carnegiea* and *Tetragonia*) form a monophyletic group. The genus *Tetragonia* sisters *Mesembryanthemum* based on our combined data (Fig 4).

Synonymous and nonsynonymous substitution patterns are valuable in gene evolution studies [29, 30]. Previous studies have shown nonsynonymous substitutions (Ka), which are less common than synonymous substitutions (Ks). Some plants, such as *Silene* [31] and Cotton [32], have Ka/Ks ratios > 1 in some genes, which indicates that some genes suggest a positive selection. Our results demonstrate substitution rates (Ka/Ks) in all cases that were substantially < 1 and Ka values that were far lower than the Ks values. Nucleotide substitution rates in chloroplast genes are generally lower than those in mitochondrial, nuclear, and plastid genes, which are under strong purifying selection [32, 33]. The average Ka/Ks values of the groups of cytochrome genes (0.057), ATP synthase genes (0.062), photosystem I genes (0.041), and photosystem II genes (0.043) were close to zero, suggesting that these groups of functional genes have been subjected to purifying selection. In contrast, the average values of the groups of ribosomal large unit genes (0.279), ribosomal small unit genes (0.316) and RNA polymerase (0.165) were higher than those of the groups of other functional genes (Fig 6 and S3 Table).

## Supporting information

S1 Table Primers used for four junctions. (DOCX)

S2 Table Studied taxa, GenBank accession numbers of reference and chloroplast genome characters. (XLSX)

S3 Table Nonsynonymous (Ka), synonymous (Ks) and Ka/Ks substitution rates of functional gene groups. (CSV)
