# Supplementary material for: Complete chloroplast genome of Tetragonia tetragonioides: Molecular phylogenetic relationships and evolution in Caryophyllales
Source: PLoS One. 2018 Jun 22;13(6):e0199626. doi: 10.1371/journal.pone.0199626 (PMC6014681; doi:10.1371/journal.pone.0199626)
Supplement: S1 Table — (DOCX) [file pone.0199626.s001.docx]

| Junctions | Primer |  | Sequence |  |
| --- | --- | --- | --- | --- |
| LSC-IRa junction | LSC82394F | 5'- | AACGCCCACGGATTTGATCA | -3' |
|  | IRa83108R | 5'- | AGAGCCGGATCTAAGCGTTG | -3' |
| IRa-SSC junction | ycf1bF | 5'- | AACAAAGAATTTCTTTTACATATCCACC | -3' |
|  | ndhFR | 5'- | ACGAATAATTGATGGAATTCCGAATGGTT | -3' |
| SSC-IRb junction | IRa124245F | 5'- | TATGAACGGCGAGGCACTTT | -3' |
|  | ycf1aR | 5'- | GGAATCGTCCAACGCGATAT | -3' |
| IRb-LSC junction | rpl2-98F | 5'- | CGGGTGCTGTAGCGAAACTA | -3' |
|  | LSC288R | 5'- | GGGCAGTACCGGTTTCTTGA | -3' |

S1 Table. Primers used for four junctions
